# How I Work Smarter: A Qualitative Analysis of Emergency Physicians’ Strategies for Clinical and Non-clinical Productivity

**DOI:** 10.7759/cureus.4499

**Published:** 2019-04-18

**Authors:** Benjamin Azan, Marilyn E Innes, Brent Thoma, Michelle Lin, Alex Van Duyvendyk, Zafrina Poonja, Teresa M Chan

**Affiliations:** 1 Emergency Medicine, Ochsner Health System, New Orleans, USA; 2 Emergency Medicine, Michigan State University College of Human Medicine, Grand Rapids, USA; 3 Emergency Medicine, University of Saskatchewan, Saskatoon, CAN; 4 Emergency Medicine, University of California San Francisco, San Francisco, USA; 5 Family Medicine, University of British Columbia, Kelowna, CAN; 6 Emergency Medicine, University of Alberta, Edmonton, CAN; 7 Health Sciences, McMaster University, Hamilton, CAN

**Keywords:** productivity, thematic analysis, efficiency, burn out, emergency medicine, communication, qualitative analysis, working smarter

## Abstract

Introduction

Emergency physicians’ (EP) clinical and professional non-clinical environments can be stressful and lead to burnout. However, some EPs thrive in these environments. To date, there is limited research investigating the strategies that successful EPs use to be maximally productive.

Methods

A snowball sampling technique was used to identify peer-nominated EPs who were, within their community of practice, subjectively felt to be successful and efficient. Participants answered a standardized set of questions addressing their efficiency patterns that were published as part of the “How I Work Smarter” blog series on the Academic Life in Emergency Medicine website. Two reviewers performed an inductive qualitative thematic analysis to code and summarize their responses and develop a thematic framework that described patterns of EP productivity.

Results

Two themes, communication and efficiency, were applicable in the clinical and non-clinical arenas. Location and environment was a major theme in the non-clinical arena. The themes task management and prioritization, tools for wellness, and motivators spanned both environments. Each theme included several strategies that were felt by the respondents to improve productivity and efficiency.

Conclusion

We described a thematic framework of productivity strategies for EPs that may increase productivity, improve work-life balance, and decrease burnout. EPs interested in increasing their efficiency both within and beyond the clinical area may consider adopting these strategies.

## Introduction

Emergency physicians (EPs) work clinically in stressful, overcrowded environments [1] and often have additional non-clinical responsibilities such as conducting scholarly work, participating in administrative initiatives, and/or teaching when not in the clinical setting. Combined, these expectations may contribute to task overload [2,3] and the high rates of burnout seen within the specialty [4,5].

Despite these challenges, some EPs thrive. Within their communities of practice, they are recognized by their peers for productivity in their personal and professional lives. We sought to develop a greater understanding of the efficiency strategies that these EPs used to function at such a high level. Unfortunately, previous productivity studies in emergency medicine have focused on department-wide efficiency [6-9] and only rarely have provided guidance on improving individual productivity [10,11].

The participants in the “How I Work Smarter” (HIWS) blog series on the Academic Life in Emergency Medicine (ALiEM) website were nominated by their peers as experts in personal productivity and efficiency. We performed a qualitative analysis of their responses to develop a thematic framework that could assist the larger population of EPs in optimizing their productivity across clinical and non-clinical environments.

## Materials and methods

The McMaster University/Hamilton Integrated Research Ethics Board granted ethical approval for this retrospective qualitative study. All participants provided written informed consent before their data was extracted for analysis.

Study participants were identified based on their authorship of posts in the publicly available HIWS online blog series published on the ALiEM website. The ALiEM website is an English-language emergency medicine education website that receives more than 1,500,000 pageviews annually. The HIWS blog series consisted of 46 posts published between July 18th, 2014 and September 13th, 2015. Each blog post in the HIWS series consisted of six introductory questions and seven productivity questions (Appendix 1). Although photos of the participants and their work spaces were also published in the HIWS blog posts, they were not included in the analysis.

**Figure 1:**
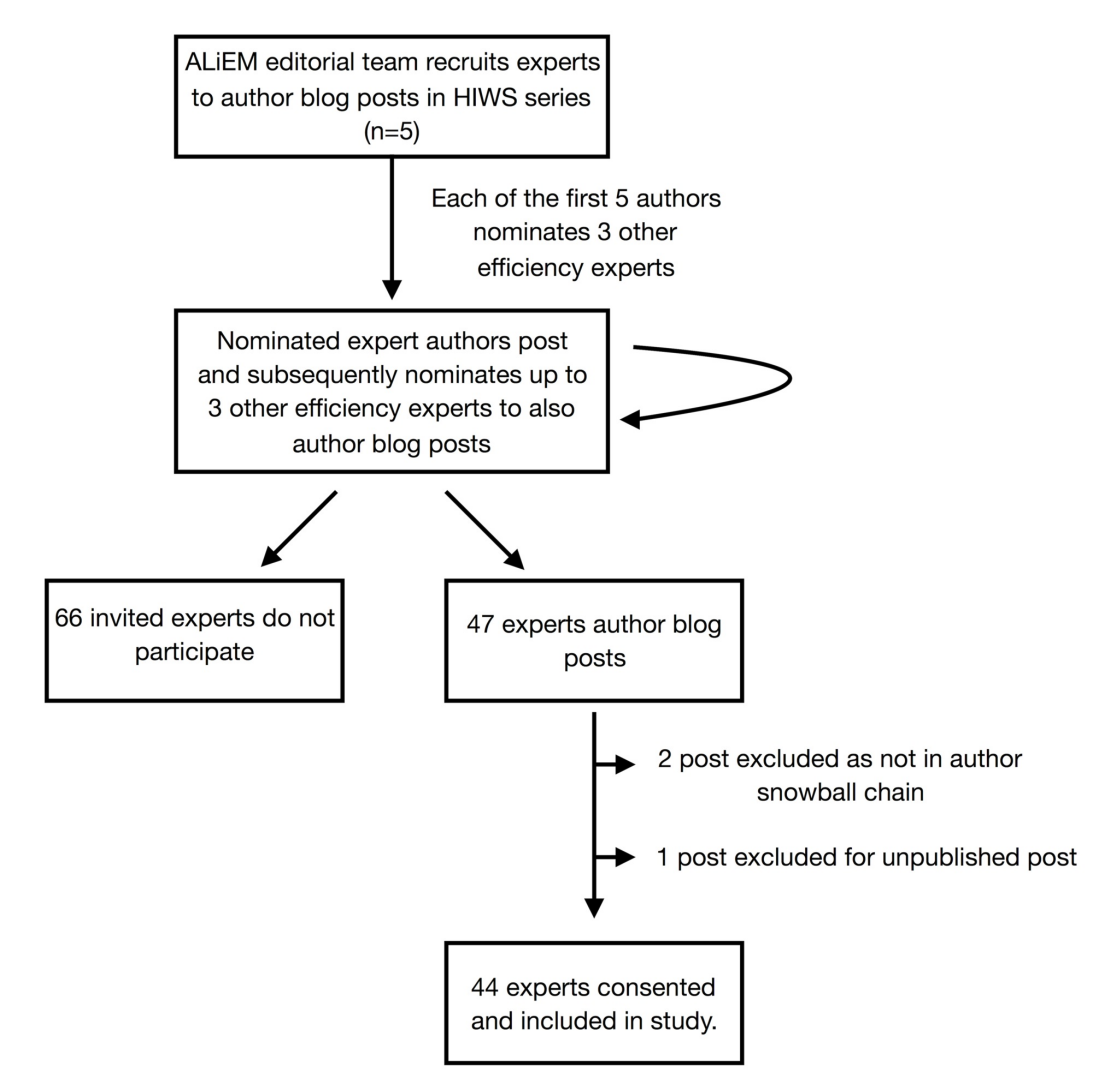
Recruitment Methodology for Identifying and Studying Efficiency Experts ALiEM: Academic Life in Emergency Medicine; HIWS: How I Work Smarter

Figure 1 depicts the participant recruitment methodology. A snowball sampling technique [12] was used to identify participants for the blog series by asking each participant “Who would you love for us to track down to answer these same questions?” The editor-in-chief of ALIEM, Michelle Lin, authored the first post. She then chose the subsequent four authors in the snowball process. Each subsequent participating expert was asked to nominate up to three peers. The focus of the blog series, interviewing exceptionally productive and efficient EPs, was made clear to all participants prior to their nominations of potential participants. However, no further definitions or objective measures of productivity/efficiency were required for nomination. HIWS posts from individuals who were not nominated in this way were excluded from the study (Figure 1). Of 46 published blog posts, 44 met inclusion criteria. Following publication of their post, participants were contacted by email to request consent to use their data in this study and for additional demographic data (Appendix 2). All 44 experts whose posts met inclusion criteria consented to participate in the study.

A qualitative analysis of the participants’ publicly available HIWS posts was performed using a constructivist grounded theory approach [13]. As no singular conceptual framework addressed the breadth of questions on productivity posed within the HIWS series, a new framework was developed. Our analytic team initially comprised of three investigators (BA, AV, MI), who reviewed the blog posts in totality, and developed a coding structure with relevant themes and subthemes.

The coders considered reflexivity while coding the data. They included a Canadian family medicine resident (AV), an American junior clinician educator and EP (BA), and a mid-career clinician educator and EP with experience working in Canada and in the United States (MI). During the coding process, they challenged each other regarding their interpretation of the dataset and discussed their own perspectives and experiences. The rest of the team (BT, ML, ZP, TC) reviewed the transcripts and acted as data auditors to ensure that the analysis was rigorous and reproducible. All meetings related to the planning and execution of the research and manuscript were held virtually via Internet video conferencing technology or telephone conversation.

Prior to coding, identifying information was redacted and each post was saved as a separate, individual document. The data were analyzed in groups of six posts. Reviewers met after the analysis of each group of six posts to compare results and discuss emerging themes. This process was repeated until reviewers felt thematic sufficiency was reached. Thematic sufficiency was felt to be reached after coding 18 posts. Extracted themes were organized into a framework. Theme structure, hierarchy, and overlap were discussed at each meeting and recorded in a separate theme document. A consensus thematic framework for the data was derived and refined with input of the entire group. A single member of the analysis team (BA) reviewed all subsequent posts against the thematic framework to ensure that no major themes were missed.

After the framework was complete, two of the reviewers (BA, MI) used it to reanalyze the entire set of blog posts. A table was constructed in Microsoft Excel (Redmond, WA, USA). Themes were placed on the X axis and blog posts on the Y axis. Two reviewers coded the entirety of each blog post separately; reviewers then met to compare coding and resolved differences by consensus. Generation of results was performed by the same two reviewers. Each reviewer analyzed the data coded to half of the framework categories. To ensure the rigor of the analysis, supporting quotations were placed within their corresponding themes. The results of the data analysis were then reviewed by two other team members (BT, TC) to ensure reproducibility.

## Results

Our conceptual framework for individual productivity is summarized in Table 1. Participant demographic data is summarized in Table 2. Major themes that emerged included communication, efficiency, environment and location, task management, prioritization, wellness, and motivators. EPs used different strategies for managing productivity in their clinical and non-clinical work settings.

**Table 1:** Conceptual Framework for Thematic Analysis of the How I Work Smarter Data

Clinical Work	Professional Non-Clinical Work
Clinical communication	Non-clinical communication
Clinical efficiency	Non-clinical efficiency
	Location and environment
Task management and prioritization	
Tools and strategies for wellness	
Motivators	

**Table 2:** Demographic Characteristics of the Study Participants

Characteristic	Participants
Total Number of Participants	44
Mean Age in Years (range)	39.5 (25-55)
Mean Number of Years in Practice (range)	11 (1-23)
Gender	
Female	15 (34%)
Male	29 (66%)
Race/Ethnicity	
Asian	5 (11%)
White/Caucasian	33 (75%)
Other	3 (7%)
Not Reported	3 (7%)
Practice Environment	
Academic	33 (75%)
Community	2 (5%)
Both Academic and Community	8 (18%)
Not Reported	1 (2%)
Mean Number of Work Hours Per Week (range)	
Clinical Work	22.9 (8-76)
Medical Education	11.8 (1-30)
Research	7.4 (0-50)
Administration	11.4 (0-60)

Communication

Clinical Communication

Clinical documentation was identified as a productivity strategy in the clinical environment. Participants viewed the documentation process as an important means of communicating their clinical reasoning to others, but also linked documentation to their cognitive processes. The act of documentation helped the clinician remember the patient and served as a check against missing an important diagnosis. For those who engaged in electronic charting, the use of macros (pre-written text) served a similar cognitive function: acting as a reminder system which prompted them to ensure that all pertinent aspects of the presentation have been addressed, while simultaneously improving documentation efficiency. Participants noted that it may be important to restrict the use of macros due to the potential for documentation error in severely ill patients or patients presenting atypically.

“My MDM [medical decision making] is how I remember the patients in my brain. Even if every back pain has ALMOST the same MDM… there’s always a little bit that’s different. And the sheer fact of typing ‘no signs/sxs of epidural abscess, cauda equina, trauma…’ makes me double-check in my brain that they really don’t have signs or symptoms of these life threatening disorders.”

The documentation process was considered especially valuable for trainees as it allowed supervisors to understand their clinical reasoning.

Multiple participants noted that the timing of charting could affect patient care. Some felt that charting during an encounter impaired the quality of the patient encounter. Others noted that a lag time between a patient encounter and charting (e.g., deferring it to the end of shift) resulted in poor quality documentation.

“I don’t document during the encounter. I’d rather have an effective, efficient patient encounter and charting session in series, rather than do both tasks simultaneously, but poorly.”

“Documentation suffers if we delay between the encounter and charting.”

Participants thought that a careful approach to history-taking saved time by avoiding repeat visits to the patient, with the added benefit of improving rapport. They also described verbalizing their cognitive processes in their conversations with patients as a method for assisting patients in ascribing value to the clinician’s cognitive work. They felt that increased communication with patients led to lower rates of diagnostic testing.

The process of communicating one’s thoughts to others was not just restricted to patients. Explaining cognitive processes aloud to colleagues was thought to help strengthen team relationships and avoid frustration.

Participants identified strategies to reduce time spent on clinical documentation. Those who documented using paper records described using name stamps and printing their notes to save time and ensure legibility. Participants advised against duplicating information documented elsewhere.

Non-clinical Communication

With regard to non-clinical communication, participants discussed detailed methods for processing and organizing email, which included timing of replies, tools for email management, and content of emails.

"'Inbox Zero' was the most common strategy for email processing [14]. It consists of a 'one touch' approach to email in which the user either immediately deletes email, replies, forwards, or flags the email for later response."

“Delete or move. The only emails in my inbox are ones that require a thoughtful response and I haven’t had time to generate one. That’s probably less than five a day. Then respond and delete.”

Participants also converted emails into tasks which they subsequently organized into folders based on either theme or urgency. Participants noted that folder organization allowed them to easily locate emails. Search was also mentioned on several occasions as an alternative or complement to organizing via folders.

“I have rigged my Gmail so that I can quickly sort my inbox immediately into actionable items of: To Do Today, To Do Soon, Scheduled for Meeting, Awaiting Reply, Non-Urgent Tasks.”

Participants described “batching” emails, an approach that involved allocating specific amounts of time to email, in order to prevent email from encroaching on other tasks. Email occurred at set times, or between other important tasks, or as a break from another cognitively heavy tasks.

“Save email time for when you need a mental break. Batch email processing, and don’t constantly check during the day.”

Email tools were mentioned by several participants. Most often mentioned were Boomerang (Mountain View, CA, USA) and ActiveInboxHq (Brighton, UK). These tools allow users to be notified when emails are not replied to, provide read receipts, or schedule email to be sent later. Others mentioned tools that incorporate voice-to-text and macros for email writing.

Participants discussed the content of email communication. The two recommendations focused on politeness and positivity, with acknowledgement that tone and intention can be lost in email communication.

Participants discussed alternative modes of professional non-clinical communication. In-person discussions and voice calls were occasionally mentioned as more efficient solutions than email.

“Know the limits of email. Some conversations need to happen in person. You will save clean up time if you better discern what is appropriate for email and not. For example, if you want to create change at work, don’t just send a complaint email.”

Efficiency

Clinical Efficiency

Practices towards improving efficiency in the clinical work setting included modification of the work environment, cognitive strategies, management of professional relationships, ergonomic strategies, resource utilization strategies, and knowledge organization strategies.

Participants advocated an active approach to identifying and modifying impediments to efficiency in the clinical work environment.

“Flow-destroying situations include task interruptions, unnecessary alarms, fights with other services, and poor physical plant/work set-ups. All these are preventable and fixable with forethought and a group of colleagues that want change.”

Others mentioned turning off phone notifications or using digital assistants to schedule reminders as methods for minimizing interruptions.

Cognitive efficiency, or the importance of thinking efficiently in the work environment, was a prominent subtheme. Participants identified that delaying decision-making was a source of inefficiency. Further, they noted that it was crucial to prioritize patient reassessment and disposition for forward patient flow.

“Always make an active decision. You should not be constipated about decision making or you will harm your patient. Sometimes the active decision might be to do nothing. That is ok - it is still a decision. On the other hand, doing nothing because you never got around to making a decision is not.”

Two participants cited the value of thin-slicing, which is the concept of making quick inferences based on limited information [15,16]. They explained that this practice allowed for cognitive offloading and increased efficiency of thought.

“The concept of thin-slicing is a way to debulk an overwhelming ED [emergency department]. Later, go back and spend time after prioritizing your thin slices.”

Multitasking strategies were discussed. Although one participant specifically mentioned that “multi-tasking is a myth”, several mentioned the benefit of concurrently managing related tasks. A recurring example was the description of concurrent task management during the patient interview or during a resident presentation.

“I find that charting at the time of interview or resident/student presentation allows for better documentation, and lets me focus more on medical decision-making.”

Strong interprofessional relationships were noted to improve clinical efficiency by allowing for better teamwork and effective delegation.

Participants also described attention to efficiency of movement. Having all the necessary tools during patient encounters was considered important in this regard, as was batching patient assessments based on geographical proximity in the emergency department. Having one comprehensive encounter with a patient, rather than multiple repeated brief encounters, was thought to lead to improved efficiency.

“Always take all the necessary tools you’ll need when examining a patient into the room with you. Sore throat? Make sure you have a tongue depressor. Abdominal pain? I bring the ultrasound in with me.”

Several participants discussed the efficiency gained by being judicious with resource utilization.

“Only order the tests you need (things that change your decision making based upon the result).”

One expert identified that personal time and resources also need to be managed to ensure that fatigue and hunger do not adversely affect efficiency.

Having a plan for organizing knowledge was also mentioned as a strategy for increased efficiency. Several participants suggested having a clear, reliable system for information retrieval during one’s clinical shifts.

“I have one favorite app … and I know where and how to search it well.”

Non-clinical Efficiency

Participants described both digital and physical work tools that they used to maximize their efficiency in the non-clinical setting.

Tools for digital collaboration, such as Slack (San Francisco, CA, USA), Google Hangout (Mountain View, CA, USA), and Skype (Palo Alto, CA, USA), were cited as important efficiency aids. Participants also valued the ability to keep the work environment digitally available and synchronized across locations via the use of cloud-based platforms.

“Using a combination of Box, Dropbox, Google Drive, and iCloud …. I am able to work on projects whenever and wherever I have the time…”

However, several participants also mentioned the need to strategically limit access to digital technology to maximize efficiency. This was discussed in the context of turning off alerts and social media in order to focus on work-related tasks.

Participants repeatedly described the benefits of having multiple computer screens as well as multiple computers to increase work efficiency. Having both a work and personal computer was frequently mentioned. Alternatively, several participants noted the strategy of having a single laptop which could be taken across work environments, thus enabling a familiar digital work environment in any location. One participant highlighted that they quarantined each type of work to an assigned computer.

Digital calendars played a central role in the efficiency of participants. They were mentioned as both a repository of upcoming activities and as the source of reminders to help stay on task. Several authors used their calendars as to-do lists.

“I block off time to achieve my “to do’s” in my calendar. If it’s not in my calendar, it is unlikely to get done.”

One participant was an outlier regarding digital technologies in that he did not utilize them. He proposed a simplified approach which consists of only a piece of paper with a task list.

“I don’t have any special method of organization except a folded piece of paper that I carry in my pocket and write notes on when something needs to be done … I don’t worry about being organized… I just do what needs to be done in the order in which things need to be done.”

Some approaches spanned the spectrum of digital and analog strategies. One example was post-it notes. Several participants mentioned their utility for visual task reminders in the work area. Their digital equivalent, sticky note software, was also mentioned.

Despite an overall clear reliance on digital tools, the use of physical non-digital based tools remained prominent. A frequently noted solution was to have a whiteboard, which tracked important data such as tasks, deadlines, and projects. One participant made an analogy about how she listed tasks on a “tracker board” and “runs this list” often in a similar manner as they would while on shift in the emergency department. Physical notebooks were also mentioned on several occasions as a casual and portable tool for organizing information, to-do lists, and ideas.

“When planning out projects and organizing my thoughts, I like to draw. I find that analog tools foster creativity. So one of my favorite purchases is a large whiteboard for my home office.”

Location and environment

Participants identified a variety of preferred work environments for their non-clinical work, including trains, gyms, outdoors, shared office space, private home offices, and private hospital or university offices. Individuals selected their locations based on a variety of factors: the nature of the task being performed, interpersonal relationships within a given environment, ability to integrate work with daily life, time of day, and the presence or absence of distracting factors. Regardless of the location, there was a clear emphasis on mobility and portability.

The nature and complexity of tasks influenced work location. Participants preferred more isolated environments (e.g., closed-door office) to complete focused, cognitively heavy tasks, while choosing collaborative, open spaces for group work. Environments like gyms, outdoors, and family kitchens were reserved for tasks perceived as less important or complex. Sometimes the nature of the task dictated the environment; for example, one participant preferred to record podcasts from the closet because the environment had better acoustics.

“I sometimes require a concentrated/classic space (desk, desktop, phone, etc.) for me to handle the administrative requirements of the jobs. However, I also need a collaborative, open-space where I can work with others and think in a more unrestricted way.”

The nature of interpersonal relationships within work environments also influenced their selection. Some participants preferred home environments as they promoted interconnectedness with family, while others identified family as a potential distractor. With regard to office environments, some participants viewed the presence of their colleagues as a source of potential inspiration or happenstance collaborations, while others saw their presence as a distraction.

Several participants chose work environments in order to integrate daily living activities with work activities. This included working while commuting or dictating e-mails while exercising. Roles and career stage also influenced the choice of work location. One trainee-level participant noted not having dedicated office space, resulting in home and coffee shop environments as default work spaces. Some attending-level participants designated formal university or hospital offices as locations for meeting with colleagues. Finally, physiological needs and personal qualities of the participants themselves were noted as influencing choice of work environment.

“I actually do most work on a spinning bike at home because I can’t concentrate well with my heart rate below 100.”

Within a given work environment, participants often noted arranging sensory input to make the environment more appealing. This included the addition of photos of family, aromas, and the presence of food and beverages.

Task management and prioritization

There were three main subthemes in this domain: identifying and selecting projects, strategies for prioritizing amongst multiple projects, and determine the timing and order of tasks. Collaboration, delegation, and time management were felt to be important skills.

Factors for selecting projects appropriately included goal alignment (i.e., how it fit with long-term goals), multipurpose reuse (i.e., a specific project could be utilized and counted multiple times), and collaborators. Participants noted that saying no to some projects allowed them to spend time on other priorities, such as other projects or family events. They also used their anticipated inability to meet deadlines as a criterion for saying no to projects. This strategy, however, was emphasized for later career stages, as saying yes in early career was seen as important for development.

“For everything that you say yes to, you have just said no to something else. Keep this in mind as you accept new projects and tasks, as the new project may take away from family life and hobbies.”

Participants used prioritization systems to manage workload. Simple systems were preferred, such as lists or dashboards. Participants also described systems for addressing important non-urgent issues as a strategy for effecting change.

Teamwork was identified as a means of improving productivity via a strategy of delegation. Participants delegated tasks that were outside of their core competencies to others in order to be more effective. Value was attributed to teams composed of cross-disciplinary, self-motivated members.

“[Allow] colleagues to support you and [permit] them to be creative as a means of becoming more efficient. . . . [T]his means that when I task people I ask them to deliver the outcome, rather than micromanage the task processes. This is much more time efficient for me and I often find that they develop their own systems which are better than your original idea.”

The concept of time was a recurring topic. Multiple time management strategies were mentioned to monitor time spent on specific tasks. Participants valued “being early” on deadlines as evidence of reliability and commitment. Actively reserving time for non-work pursuits was also deemed important.

“[O]nly allow a certain amount of time for each task. When that time is up, move onto the next task regardless.” 

Some participants advocated beginning tasks without substantial planning (e.g., “just do it”). Others described delaying tasks until they had adequate time available to complete the task. Some reported difficulty in accurately anticipating the amount of time needed for some tasks.

Tools and strategies for wellness

Participants reported personal wellness, participation in recreation, and family activities as a catalyst to their overall efficiency. Multiple strategies for attaining wellness were discussed including maintaining interpersonal relationships, balancing demands of work and personal life, participating in work activities that result in feeling of enjoyment, and prioritizing physical wellness.

Interpersonal relationships with family and co-workers were considered important. Participants felt that encouragement from family allowed them to reach their maximum potential in the work environment. Friendly and collaborative relationships with colleagues were also seen as keys to wellness and achievement.

Participants thought that balancing personal life and work allowed for improved wellbeing and career longevity. Several participants mentioned the importance of setting limits or compartmentalizing as a way for controlling work demands. One participant felt that true work-life balance does not exist and that some degree of imbalance must be accepted.

“Figure out what means the most to you in your life, and schedule those things in first, along with things that are necessary for your health and well-being (sleep, family time, exercise, etc.)"

Wellness was also noted to be influenced by work activities themselves. Participants thought work activities should result in feelings of enjoyment and a re-prioritization should be undertaken if they do not. Negative emotions at work were noted to carry into the home environment, further stressing the importance of finding work environments which promotes wellbeing.

Physical exercise and regular intake of food and fluids was also noted as important to physical wellness and efficiency. Self-care, and thus the prioritization of wellness, was felt to be the responsibility of the individual physician themselves.

Motivators

Multiple participants identified being motivated by the people with whom they collaborated. For example, helping others to succeed and grow their careers was considered to be rewarding.

“Help other people and focus on growing their careers ... I have found that my career happiness has increased exponentially by helping others.”

Passion was cited as an important factor for both the enjoyment of work and for influencing the decision to do a certain type of work. Further, passion for one’s work was associated with feelings of increased energy, focus, and success and consequently decreased burnout. Conversely, one participant mentioned boredom as a motivator, and perceived work as a mechanism to void boredom.

“I find that people who really have a passion for something will end up being successful. You have to love what you do every day to be consistently good at it and maintain that drive without burning out.”

Finally, participants noted being motivated to work efficiently in order to direct time toward other pursuits. In this context, a desire to spend more time with family was often cited.

## Discussion

In the HIWS series, peer-identified, productive EP participants described diverse strategies to optimize clinical and non-clinical productivity and efficiency. Six central themes were identified in our qualitative analyses of these expert responses. In light of concerns regarding physician burnout [5], we hypothesize that incorporation of these efficiency strategies have the potential to reduce working time, decrease stress, and reduce EPs’ risk of developing burnout symptoms.

Much of the previous literature on clinical productivity described system-based approaches rather than individual-based strategies [6-9]. Few articles have formally examined clinical productivity best practices among practitioners in the emergency department [10,11]. While our methodology was more formal, many of the themes identified in our study overlap with those found in the literature. Embracing technology, minimizing interruption, efficiency of movement, resource utilization, delegation, and prioritization are strategies identified in our study and a previous paper [10]. A recent study [11], which aimed to identify high efficiency practices in the emergency department, found that high efficiency clinical practices included carrying an average patient load, running the board, frequent touch-point conversations with team members, and calling team members by name. While average patient load is not captured in our data, the other efficiency practices described in that study were also found in our data. We believe our study adds significantly to the limited literature on clinical efficiency and is the first that we are aware of which also describes efficiency strategies for EPs in the non-clinical setting. Further, it is the first study to incorporate a participant selection strategy geared to selecting high-efficiency participants.

Mentorship and teamwork have been identified as helpful in fostering academic success [17,18], while also being protective against workplace stressors and burnout [19]. Our data identified that EPs perceive themselves as functioning within a team environment in the clinical setting. In contrast, non-clinical work seemed to be more of a solitary experience. Participants’ preference to work in non-centralized work environments, such as the home, coffee shops, and aboard transportation, may limit opportunity for interaction with academic colleagues and serve to make emergency medicine academic work a more isolating experience than we would have predicted. However, participants’ use of digital work place equivalents (e.g., Google Hangouts or Skype) may mitigate this effect. We hypothesize that an EP’s largely asynchronous lifestyle may also be a barrier to the establishment of physical proximity to other EPs during their non-clinical work hours. Participants did, however, identify that choice of teammates was important when selecting non-clinical projects. It is possible that the desire to mentor or receive mentoring was integrated into those decisions.

Participants’ responses were frequently contradictory. For example, some preferred digital technology while others preferred paper-based methods. Some preferred focusing on single clinical tasks while others advocated multitasking. While best practices did emerge, the lack of agreement within many of our data’s themes illustrates that no single approach is best for all practitioners. Personal characteristics such as type of practice, location, age, and family situation undoubtedly affect which efficiency practices are best suited for each individual. Further, concept of efficiency likely had differing interpretations by study participant, resulting in variable strategies emerging from the data. Our study suggests that approaches should be adapted to one’s own unique situation.

This study utilized a novel approach to recruit and publish insights on efficiency from a blog-based platform that were ultimately amalgamated through a qualitative analysis. This approach resulted in responses from many prominent, busy EPs, who we hypothesize would likely have been reluctant to complete a traditional, time-intensive qualitative survey. They wrote eloquent and detailed blog posts outlining their experiences in this HIWS series, potentially being drawn to this format as an engaged citizen of emergency medicine’s virtual community of practice [20]. Additionally, the previous public identification of prominent participants likely increased the credibility of the initiative and therefore the willingness of others to participate.

Limitations

Our study focused on a single specialty, which limits the transferability of our findings to other groups. However, participants included physicians of varying age, career stage, nationality, and practice location, representing diverse perspectives within emergency medicine.

Despite perceiving thematic sufficiency after the analysis of 18 posts, additional posts were coded resulting in continued minor modification to the thematic structure. This suggests that sufficiency may not have been reached, even after analysis of the entire set of posts. Had more than 44 participants contributed to the data set, new themes or efficiency practices could have emerged. This likely reflects the complex interaction between productivity strategies and other factors such as work environment, health systems characteristics, and personal characteristics. Further, it is not proven whether the productivity strategies outlined in the study truly correlate with efficiency. Despite participants being seen as productive by their peers, the study did not objectively measure participant’s efficiency.

The question set presented to the participants was not designed with a research project in mind. Several questions asked to participants (Appendix 1) were focused, and likely led participants to target their responses more heavily toward certain aspects of their work than they might have, had the questions been more open ended. This may have led to an over-representation of certain productivity strategies in the data.

## Conclusions

Strategies used by EPs to maximize individual productivity in clinical and non-clinical work have not been well described in the literature. We conducted a qualitative analysis of a publicly available blog post set to identify productivity strategies used by peer nominated EPs. Our results suggest that EPs use a variety of strategies across work settings to improve clinical as well as non-clinical output and to support their desire to maintain balance between work and personal lives. The insights found within this thematic analysis should be helpful for EPs who hope to accomplish more within their working hours.

## Appendices

Appendix 1

Survey Questions Sent to Nominated Participants

Introductory Questions

Name

Location

Current job

One word that best describes how you work

Current mobile device

Current computer

Productivity Questions

What’s your office workspace setup like?

What’s your best time-saving tip in the office or home?

What’s your best time-saving tip regarding email management?

What’s your best time-saving tip in the Emergency Department (ED)?

ED charting: Macros or no macros?

What’s the best advice you’ve ever received about work, life, or being efficient?

Is there anything else you’d like to add that might be interesting to readers?

Snowball Question

Who would you love for us to track down to answer these same questions?

Appendix 2

Demographics Survey Sent to Participants at the Initiation of the Study

Age

Years in practice

Race/ethnicity that you identify most with:

o   American Indian, Alaska Native, Australian Native

o   Asian

o   Black or African American

o   Native Hawaiian or Other Pacific Islander

o   White or Caucasian

o   Other (specify)

o   Multiracial

Number of children

Scope of your practice:

o   Academic

o   Community

o   Both

How many hours a week on average do you spend on the following activities:

o   Clinical time

o   Medical education

o   Research

o   Administration

o   Other ​​
